# Identification of Key Factors for Anoxic Survival of *B. cenocepacia* H111

**DOI:** 10.3390/ijms23094560

**Published:** 2022-04-20

**Authors:** Sarah Paszti, Alessandra Vitale, Yilei Liu, Rubina Braunwalder, Ratchara Kalawong, Olivier Biner, Gabriella Pessi, Leo Eberl

**Affiliations:** Department of Plant and Microbial Biology, University of Zurich, Zollikerstrasse 107, 8008 Zürich, Switzerland; sarah.paszti@uzh.ch (S.P.); vitale.aless@gmail.com (A.V.); yilei.liu@uzh.ch (Y.L.); rubina_braunwalder@hotmail.com (R.B.); ratchara.kalawong@botinst.uzh.ch (R.K.); olivier.biner@botinst.uzh.ch (O.B.)

**Keywords:** *Burkholderia cenocepacia* H111, *Bcc*, cystic fibrosis, Tn-seq, anoxic survival, *Galleria mellonella*, virulence factors

## Abstract

*Burkholderia cenocepacia* is an opportunistic pathogen that can lead to severe infections in patients suffering from cystic fibrosis (CF) and chronic granulomatous disease. Being an obligate aerobe, *B. cenocepacia* is unable to grow in the absence of oxygen. In this study, we show that the CF isolate *B. cenocepacia* H111 can survive in the absence of oxygen. Using a transposon sequencing (Tn-seq) approach, we identified 71 fitness determinants involved in anoxic survival, including a Crp-Fnr family transcriptional regulatory gene (*anr_2_*), genes coding for the sensor kinase RoxS and its response regulator RoxR, the sigma factor for flagella biosynthesis (FliA) and subunits of a cytochrome *bd* oxidase (CydA, CydB and the potentially novel subunit CydP). Individual knockouts of these fitness determinants significantly reduced anoxic survival, and inactivation of both *anr* copies is shown to be lethal under anoxic conditions. We also show that the two-component system RoxS/RoxR and FliA are important for virulence and swarming/swimming, respectively.

## 1. Introduction

Despite recent improvements in treatment regimens, pulmonary disease remains the leading cause of morbidity and mortality in patients with cystic fibrosis (CF) [1]. The pathophysiology of this autosomal recessive inherited disease is based on a single mutation in the cystic fibrosis transmembrane conductance regulator gene (CFTR), resulting in the thickening of mucus [2,3], which becomes prone to colonization by opportunistic pathogens. Chronic infection can lead to bronchiectasis (lung damage) and respiratory failure, often with deadly outcomes [4]. The local oxygen concentrations in the CF lung range from aerobic to micro-oxic, and even anoxic pockets in mucus plugs or in deep biofilm layers have been reported [5,6]. Thus, bacteria colonizing the CF mucus must be able to adapt their metabolism to different oxygen levels. Indeed, it was shown that the facultative anaerobe *Pseudomonas aeruginosa*, the predominant infectious agent invading the airways of CF patients [7], is able to grow in oxygen-limited environments by using a combination of micro-oxic respiration, denitrification and arginine and pyruvate fermentations [5]. Denitrification, which is the complete reduction of nitrate (NO_3_^−^) to molecular nitrogen (N_2_), is an important alternative energy-conserving pathway used in the absence of oxygen. The energy yields of denitrification consist of approximately nine ATPs per two molecules of nitrate [8] in contrast to the 36 ATP produced per one glucose consumed in aerobic respiration. In the absence of oxygen and nitrate, *P. aeruginosa* is able to ferment arginine to ornithine, thereby generating one molecule of ATP per molecule of arginine via substrate-level phosphorylation [9]. *P. aeruginosa* is also able to survive anoxic conditions by producing lactate via a mixed-acid fermentation of pyruvate [10]. Another important anoxic survival mechanism is the reduction of phenazines, small excreted molecules capable of extracellular electron transfer, which can be coupled to ATP generation. *P. aeruginosa* reduces phenazines, which then get oxidized by oxygen, other oxidants, or by *P. aeruginosa* [11,12]. CF patients suffering from *P. aeruginosa* infections are often co-infected with other opportunistic pathogens. Of particular concern are infections with strains belonging to the *Burkholderia cepacia* complex (*Bcc*), which can cause a life-threatening systemic infection known as *cepacia* syndrome [13]. The *Bcc* comprises more than 25 closely related species [14], and their prevalence in CF patients is between 2 and 4% [15]. Members of the *Bcc* are highly versatile and adaptable and have been isolated from diverse habitats such as soil ecosystems (plant rhizosphere), aquatic environments and infected humans [14,16,17]. *B. cenocepacia* is an obligate aerobic opportunistic pathogen of the *Bcc* group that requires an oxygen concentration of at least 0.1% for growth [18]. Specific high oxygen affinity cytochrome *c* oxidases, known as cytochrome *cbb_3_* oxidases, which play important roles in the adaptation to hypoxic conditions, are almost exclusively found in *Proteobacteria* [19,20]. However, no genes coding for cytochrome *cbb_3_* oxidases were identified in the genus *Burkholderia* [18]. Moreover, *B. cenocepacia* H111 is unable to denitrify due to a lack of the gene clusters coding for enzymes required for the reduction of nitrate to dinitrogen, such as the nitrate reductase gene cluster (*nar*), the nitric oxide reductase (*nor*) and the nitrous oxide reductase (*nos*) cluster. However, *B. cenocepacia* H111 contains a cluster that potentially encodes the nitrite reductase (*I35_RS24165-24180*). The only *Burkholderia sensu stricto* species capable of denitrification belong to the *Burkholderia pseudomallei* complex (*Bpc*) [21]. A previous study showed that in the *Bpc* strain *Burkholderia thailandensis* E264, the first step in the denitrification process, namely nitrate reduction, generates sufficient energy for anoxic growth [21]. A global gene expression analysis of *B. cenocepacia* J2315 under nine different growth conditions, including growth in an environment with reduced oxygen (6% O_2_), identified a specific locus carrying genes involved in central metabolism, stress response and transport that was highly up-regulated under micro-oxic conditions. This low-oxygen-activated locus (*lxa*) was shown to be involved in the anoxic persistence of *B. cenocepacia* strains J2315 and K56-2 [22]. However, the absence of the *lxa* locus in *B. cenocepacia* H111 suggests that this strain uses other strategies to survive in the absence of oxygen.

In this study, we show that *B. cenocepacia* H111 is able to survive without oxygen and applied a high-throughput parallel sequencing approach based on Tn-seq in order to identify fitness determinants for anoxic survival. We identified a total of 71 fitness determinants for anoxic survival, which include genes encoding regulatory proteins such as Anr and the two-component regulatory system RoxS/RoxR. Deletion of both anr copies in *B. cenocepacia* H111 completely abolished the ability to survive in the absence of oxygen, and mutation of the *roxS*, the cytochrome *bd* oxidase *cydA* and the *fliA* mutant, significantly reduced survival relative to the wild-type by 10%, 25% and 40%, respectively. Furthermore, we show that RoxS/RoxR is important for the virulence of *B. cenocepacia* H111 and that the FliA sigma factor is essential for swarming and swimming

## 2. Results

### 2.1. Identification of Potential Fitness Determinants for the Survival of B. cenocepacia H111 in Anoxic Condition

**Figure 1:**
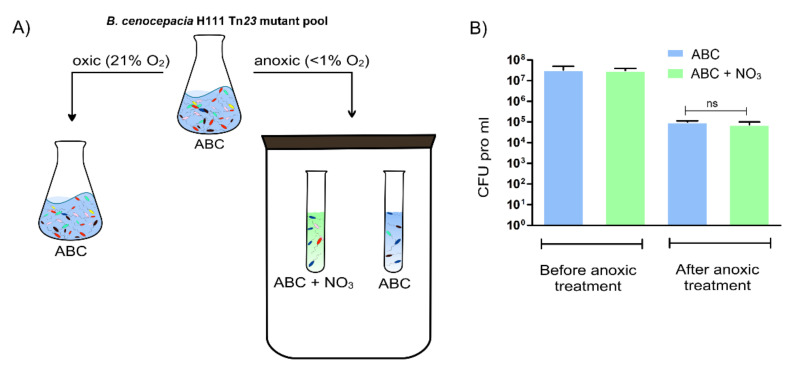
Overview of the preparation of the *B. cenocepacia* H111 samples used for Tn-seq analysis. (**A**) The *B. cenocepacia* Tn*23* mutant library was used to inoculate the control sample (grown aerobically until an OD_600_ of 0.5 at 37 °C in ABC) and the test samples (anoxically for 5 days at 37 °C in ABC or ABC with 10 mM NaNO_3_). Control and test samples were prepared for Tn-seq analysis, as described in Material and Methods. Different bacterial mutant strains are indicated with different colors. (**B**) Colony forming units (CFU) mL^−1^ at the start and end of the anoxic survival in ABC and ABC with 10 mM NaNO_3_ for *B. cenocepacia* H111 wild-type. Error bar = standard deviation (SD), ns = not significant, unpaired *t*-test (two-tailed).

The genome of *B. cenocepacia* strain H111 consists of three replicons with a total of roughly 8 million base pairs encoding for approximately 6700 proteins [23]. Previously, our lab constructed a *B. cenocepacia* H111 Tn*23* transposon mutant library [24], which was utilized in this study to determine fitness determinants important for anoxic survival of *B. cenocepacia* H111 in ABC minimal medium with and without nitrate, the latter to take the possibility of yet unidentified nitrate reductases in this organism into account. Aerobically grown *B. cenocepacia* H111 precultures were diluted in fresh ABC medium with and without nitrate and incubated for five days at 37 °C in the absence of oxygen, which was followed by an aerobic enrichment step to reach the cell densities required for Tn-seq analysis. Aerobic control samples were grown in ABC in the presence of oxygen for five generations (Figure 1A). The presence of nitrate in the growth medium did not enhance the capacity of *B. cenocepacia* H111 to survive without oxygen (Figure 1B).

**Figure 2:**
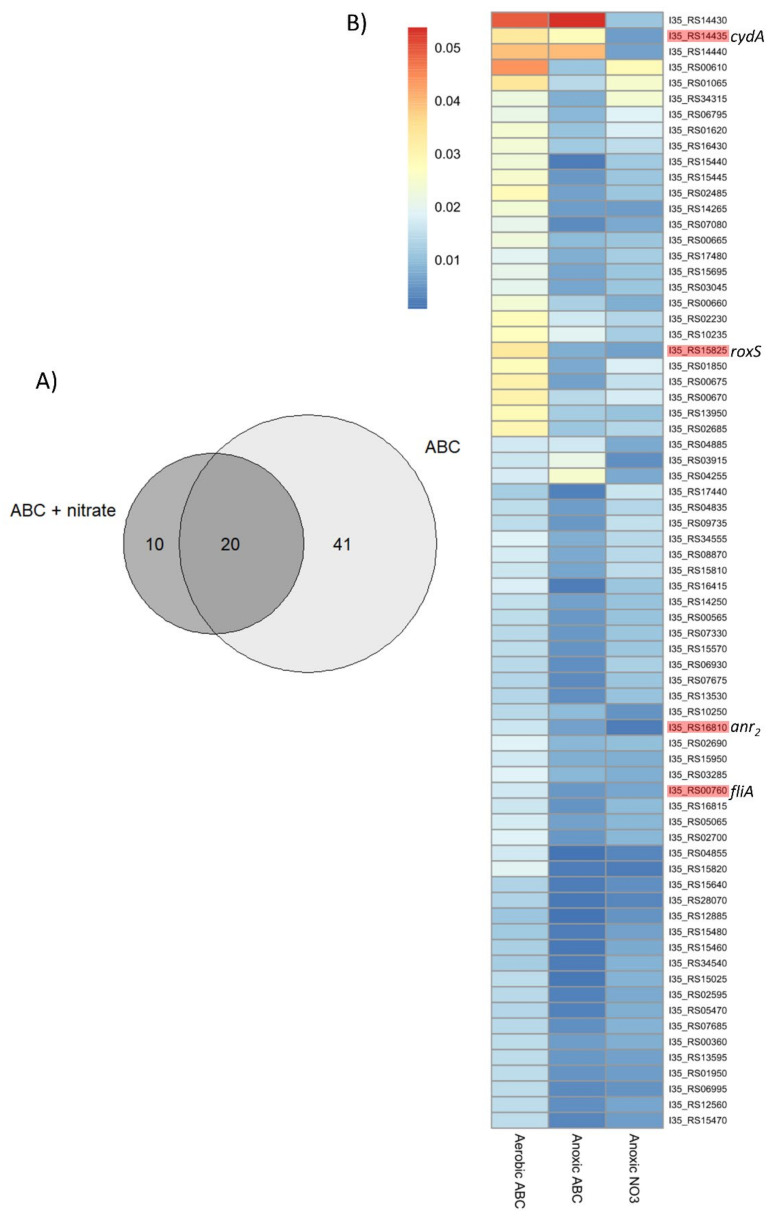
Tn-seq fitness analysis. (**A**) Venn diagram of unique and shared potential fitness determinants for the anoxic survival in ABC and ABC supplemented with 10 mM NaNO_3_. (**B**) Heat map based on the unique insertion density (UID) of the 71 potential fitness determinants in anoxic condition (ABC or ABC with 10 mM NaNO_3_). Scale shows the values of the UID for the 71 fitness determinants as a color scale. Genes highlighted in red were selected for targeted gene deletions, and their respective gene name is indicated on the right-hand side.

After sequencing and mapping the transposons sites against the *B. cenocepacia* H111 genome, a fitness analysis was performed, in which the transposon insertion abundance in the control sample (aerobic in ABC medium) was compared to the transposon insertion abundance of the anoxic test sample (anoxic in ABC with or without nitrate). If the insertion frequency significantly decreases in the anoxically grown test sample compared to the aerobic sample, the gene is important under the experimental conditions and is considered a potential fitness determinant. For the aerobic control sample and the anoxic test sample in ABC medium, we obtained approximately one million unique insertions counts (UIC), whereas, for the anoxic sample in ABC with nitrate, 840,000 UICs were mapped (Table 1). When applying the thresholds as detailed in the Material and Methods, we identified a total of 61 and 30 potential fitness determinants for the anoxic survival of *B. cenocepacia* H111 in ABC and ABC with nitrate, respectively (Table 1). Comparison of the two anoxic data sets showed that 20 determinants were shared between both conditions, whereas 41 were unique for anoxic survival in ABC and 10 were only found to be important for the anoxic survival in ABC with nitrate, resulting in a total of 71 anoxic fitness determinants (Figure 2A, Tables S2 and S3).

The anoxic fitness determinants identified in ABC and ABC with nitrate were classified into a cluster of orthologues groups (COG) by using the software EggNOG 4.5.1 [25]. After performing the Fisher tests, an overrepresentation of genes belonging to the COG category N (cell motility) was observed among the genes important for anoxic survival (Figure S1). Most of the 20 shared fitness determinants, between both anoxic conditions (Figure 2A and Table 2), were either assigned to the categories of cell motility (COG: N), transcription (COG: K) or signal transduction (COG: T).

Strikingly, 7 out of the 20 shared anoxic fitness determinants were annotated as regulatory genes (COG K or T). For example, *I35_RS16810* is an ortholog of *B. cenocepacia* J2315 *BCAM0049*, which codes for a Crp-Fnr family transcriptional regulator. Additionally, a two-component regulatory system consisting of the two genes *roxR* (*I35_RS15820*), coding for the response regulator, and *roxS* (*I35_RS15825*), encoding the sensor kinase, were identified. Another regulatory gene found in the list of the 20 shared anoxic determinants was *fliA* (*I35_RS00760*), encoding a sigma factor orthologous to the *B. cenocepacia* J2315 BCAL0144, the flagellar biosynthesis sigma factor 28. Besides *fliA*, the gene *flhC* (*I35_RS00665*) coding for another transcriptional regulator was identified. FlhC forms a complex with FlhD (FlhDC) and acts as a positive transcriptional activator of the *fliA* gene expression [26]. However, *flhD* (*I35_RS00660*) was not identified as a shared fitness determinant for the anoxic survival of *B. cenocepacia* H111 but as a potential fitness determinant under anoxia in ABC with nitrate. A gene located upstream of the Crp-Fnr regulator encodes a universal stress protein I35_RS16815, which appears to be important in both anoxic conditions.

**Figure 3:**
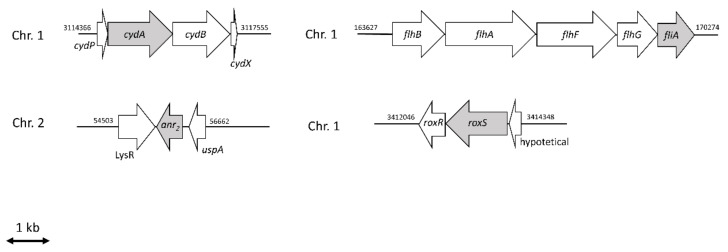
Genomic localization of the genes *cydPABX* encoding a cytochrome *bd* oxidase, *anr*_2_ coding for a Crp-Fnr type regulator, *fliA* encoding the flagellar sigma factor and the *roxS/roxR* regulatory genes. The genes that have been mutated are highlighted in grey. Chr. = chromosomal localization. Numbers refer to the genomic coordinates.

We also identified genes unique for survival in ABC (41 genes, Figure 2A, Table S2) or ABC with nitrate (10 genes, Figure 2A). Interestingly, 3 out of the 10 genes only found in ABC with nitrate are part of the same operon (COG C, Figure 2B, Table S3). The cluster (*I35_RS14430-I30_RS14440*) contains two genes coding for subunits of a cytochrome *bd* oxidase (*cydA*: *I35_RS14435* and *cydB*: *I35_RS14440*), which are preceded by the gene coding for the putative small membrane protein CydP (*I35_RS14430*) (Figure 3). 

Among the anoxic fitness determinants identified only in the absence of nitrate, we found an AraC family transcriptional regulator (I35_RS17480) and a formyltetrahydrofolate deformylase (*purU*, I35_RS14250), which catalyzes the hydrolysis of 10-formyltetrahydrofolate to formate and tetrahydrofolate in the purine metabolism [28].

### 2.2. Anr Is Essential for the Survival of B. cenocepacia H111 under Oxygen Depletion

We identified the regulatory gene *I35_RS16810* (coding for a Crp-Fnr family transcriptional regulator) as a potential fitness determinant for the anoxic survival of *B. cenocepacia* H111 in the presence and absence of nitrate. A protein sequence homology search revealed that I35_RS16810 has a sequence identity of 43% to the *P. aeruginosa* PAO1 Anr (PA1544). A search into the *B. cenocepacia* H111 genome revealed that *I35_RS16810* is paralogous to *I35_RS23120*, which is also located on chromosome 2. Alignment of the two regulatory proteins showed a 97% coverage and 53% identity [29]. From here on, we refer to *I35_RS16810* as *anr_2_* and to *I35_RS23120* as *anr_1_*. Despite their high sequence conservation, only *anr_2_* was identified by our Tn-seq analysis (Table 2).

**Figure 4:**
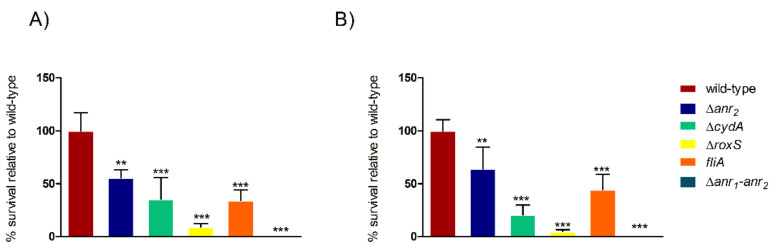
Anoxic survival of *B. cenocepacia* H111 wild-type and various mutants in ABC (**A**) and ABC with 10 mM NaNO_3_ (**B**). Y-axis shows the normalized survival in % relative to the wild-type survival according to the start and end number of CFU. Three biological replicates (*n* = 3) were performed for each survival experiment. The SD is shown by the error bars. A non-matching paired one-way ANOVA (Dunnett) was performed with confidence interval of 99%. *** = *p*-value < 0.001. ** = *p*-value < 0.01.

In order to further assess the importance of *anr_2_* for anoxic survival, we constructed a deletion mutant strain (∆*anr_2_*) and tested it for anoxic survival. The ∆*anr_2_* strain was still able to survive but to a significantly lower extent compared to the *B. cenocepacia* H111 wild-type in ABC (55% survival relative to the wild-type) and in ABC with nitrate (64% survival relative to the wild-type) (Figure 4A,B). When both *anr* regulators (∆*anr_1_-anr_2_*) were deleted, the cells were unable to survive anoxic conditions (Figure 4A,B). However, the ∆*anr_1_-anr_2_* double mutant was not affected in growth under aerobic and micro-oxic conditions in minimal and rich media relative to the wild-type strain (data not shown). 

As mentioned above, both components of a two-component regulatory system (*roxR* and *roxS*) were identified as fitness determinants for survival under anoxic conditions (Table 2). A homology search revealed that I35_RS15825 and I35_RS15820 of *B. cenocepacia* H111 shared 34% and 52% of the protein identity with RoxS (PA4494; 94% coverage) and RoxR (PA4493, 98% coverage) of *P. aeruginosa* PAO1, respectively. Accordingly, we named the I35_RS15820-25 *B. cenocepacia* two-component system RoxR/RoxS. To validate the Tn-seq results, a Δ*roxS* mutant was constructed and tested for survival under anoxic conditions. Viability of the Δ*roxS* mutant was reduced by 91% and 95% relative to the wild-type in ABC and ABC with nitrate, respectively (Figure 4A,B).

The cluster encoding a cytochrome *bd* oxidase (*cydAB*, Figure 3) was found to be important for anaerobic survival in the presence of nitrate (Figure 2A). The subunits CydA and CydB of *B. cenocepacia* H111 are highly homologous to the corresponding subunits of the *E. coli* K-12 cytochrome *bd* oxidase (CydA: 98% coverage, 68% identity and CydB: 100% coverage and 54% identity) [30]. However, the cytochrome *bd* oxidase of *E. coli* contains an additional and essential subunit called CydX [31], which was not annotated in the *B. cenocepacia* H111 reference genome. However, when the *E. coli* CydX sequence was blasted against the *B. cenocepacia* H111 genome, a *cydX* homolog was identified in the cytochrome *bd* oxidase operon (Figure 3). Interestingly, the gene coding for the small membrane protein (*I35_RS14430*), identified as a fitness determinant by Tn-seq, that is upstream of *cydA* was not found in the *cydABX* operon of *E. coli* or the *cioAB* operon of *P. aeruginosa*. A BLAST search showed that this gene is highly conserved within the order of *Burkholderiales* but not found outside of it. We name it here *cydP* as it is a putative subunit of the *B. cenocepacia* cytochrome *bd* oxidase. Next, we tested if the cytochrome *bd* oxidase is essential for survival under anoxic conditions by creating a deletion mutant of the cytochrome *bd* oxidase subunit CydA (Δ*cydA*). Relative to the wild-type, only 35% of the Δ*cydA* mutant cells survived in ABC and 21% in ABC with nitrate (Figure 4). 

For validation of the importance of the gene encoding the FliA sigma factor for anoxic survival, an insertional mutant was constructed. The *fliA* insertional mutant showed reduced anoxic survival (35% and 45% relative to the wild-type in the absence or presence of nitrate, respectively) (Figure 4). 

### 2.3. RoxS Is Required for Pathogenicity in a Galleria Mellonella Infection Model

**Figure 5:**
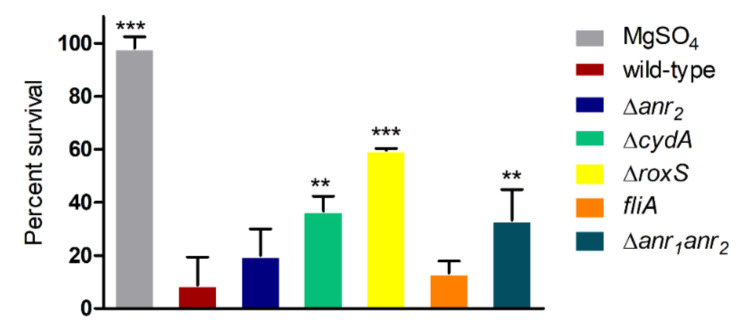
Virulence of *B. cenocepacia* H111 strains in *G. mellonella* after 48 h. Y-axis, % of survival. At least three biological replicates were performed with 10 *G. mellonella* for each sample (wild-type *n* = 9, Δ*roxS n* = 7, MgSO_4_ *n* = 5, all other samples *n* = 3). Error bars represent SD, one-way ANOVA was performed (Dunnett) with confidence interval of 99%, *p*-value ** < 0.01, *** < 0.001.

Since anoxic survival is a strategy used by several opportunistic pathogens to establish chronic infections in their host, the fitness determinants identified in this study were tested for their virulence in a *G. mellonella* infection model [32,33]. *G. mellonella* larvae were injected with approximately 2 × 10^5^ cells, and their ability to survive was monitored over 72 h (Figure 5). The Δ*roxS* mutant was most strongly attenuated (60% survival), followed by the Δ*cydA* mutant (36% survival), Δ*anr_1_-anr_2_* double mutant (33% survival) and the *anr_2_* mutant (20% survival) (Figure 5). By contrast, the *fliA* mutant killed *G. mellonella* as efficiently as the wild-type (Figure 5). 

### 2.4. The Importance of FliA for Swarming and Swimming Motilities in B. cenocepacia H111

**Figure 6:**
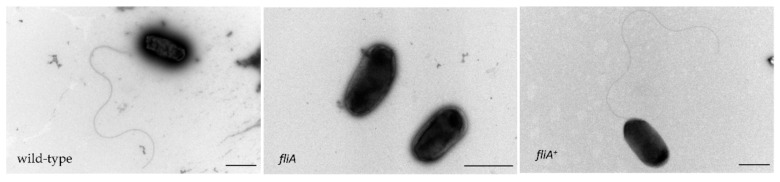
Transmission electron microscopy images of *B. cenocepacia* H111 wild-type, a *fliA* insertional mutant and a complemented strain *fliA*^+^. Two biological replicates were performed. FEI Tecnai G2 Spirit TEM (FEI, Hillsboro, OR, USA) at 120 kV acceleration voltage with the detector side-mounted digital camera Gatan Orius 1000 (4 k × 2.6 k pixels) was used. Scale bars represent 1 µm.

*I35_RS00760*, identified here as a potential fitness determinant for the anoxic survival of *B. cenocepacia* H111, encodes a protein that is similar to the *P. aeruginosa* PAO1 sigma factor FliA (PA1455) (47% identity and 59% coverage). In *P. aeruginosa,* FliA is involved in the regulation of the biosynthesis of flagella [34], and recent studies suggested that FliA is not only involved in motility but also in virulence [35]. *B. cenocepacia* H111 *fliA* is located within a cluster of genes required for flagella biosynthesis (*flhABGF*, Figure 3) [27]. *FliA* mutants of *P. aeruginosa* were reported to be non-flagellated and consequently are non-motile [35]. Transmission electron microscopy was performed to investigate flagellation of the wild-type, the *fliA* mutant and the *fliA* complemented strain (*fliA*^+^) (Figure 6). Whereas *B. cenocepacia* H111 wild-type and the complemented *fliA*^+^ strain possessed one flagellum on the pole (monopolar), the mutant was non-flagellated. 

**Figure 7:**
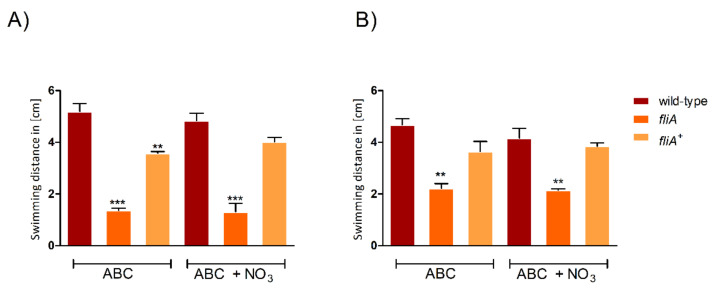
Swimming in aerobic (**A**) and micro-oxic (**B**) conditions of *B. cenocepacia* wild-type, a *fliA* mutant and the complemented strain (*fliA*^+^) in ABC or in ABC with 10 mM NaNO_3_ after 24 h. Y-axis = swimming distance in cm. Three biological replicates were performed. Error bars = SD, *p*-value ** < 0.01, *** < 0.001).

The *fliA* mutant showed reduced swimming ability in all tested conditions (aerobic, micro-oxic, with and without nitrate) compared to the wild-type strain. This defect was restored in the *fliA*^+^ complemented strain (Figure 7).

**Figure 8:**
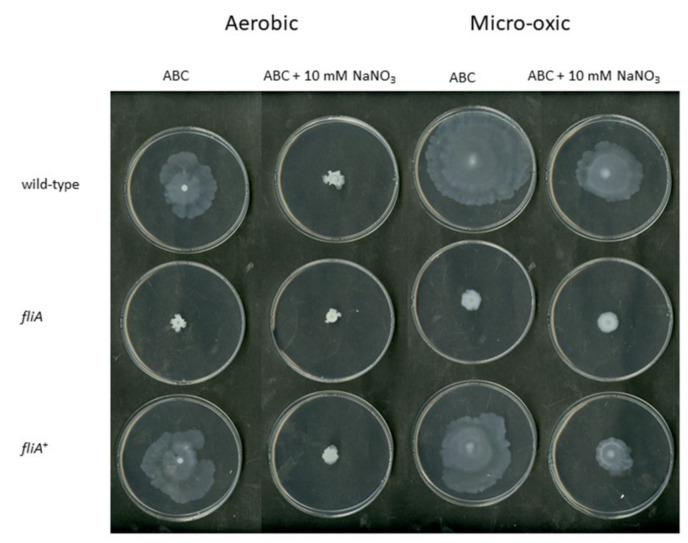
Swarming in aerobic and micro-oxic conditions of *B. cenocepacia* wild-type, a *fliA* mutant and the complemented strain (*fliA*^+^) in ABC or in ABC with 10 mM NaNO_3_ after 72 h. Three biological replicates were performed, which resulted in the same phenotype as shown above.

The *B. cenocepacia* H111 fliA mutant was also not able to swarm in any of the tested conditions (aerobic, in micro-oxic condition, with and without nitrate). However, the swarming phenotype was rescued in the *fliA*^+^ complemented strain (Figure 8). However, during the characterization of the fliA mutant strain, we realized that the swarming of *B. cenocepacia* H111 wild-type was (i) inhibited by the presence of nitrate in aerobic and micro-oxic conditions and (ii) increased in micro-oxic conditions (Figure 8). 

We also tested the strains for biofilm formation in the ABC medium in the presence and absence of nitrate. We observed a 26% reduction in biofilm formation with the *fliA* mutant (unpaired *t*-test, *p*-value < 0.0001) compared to the wild-type in the presence of nitrate, whereas in the absence of nitrate, biofilm formation in the *fliA* mutant was reduced by 16% relative to the wild-type (unpaired *t*-test, *p*-value < 0.0194) (Figure S2). The defect in the biofilm was restored to wild-type levels in the complemented strain *fliA*^+^ (Figure S2).

## 3. Discussion

In the absence of oxygen, energy can be generated by either fermentation or reduction of alternative electron acceptors such as nitrate and sulfate. In the genus *Burkholderia,* only specific strains from the *Bpc* are able to grow in the absence of oxygen by performing denitrification, i.e., reducing nitrate into dinitrogen [21], and most other *Burkholderia* species are considered obligate aerobes [22]. *B. cenocepacia* H111 is able to grow at a low oxygen concentration of 0.1%, and we show here that it is able to generate enough energy to survive in the absence of oxygen, an environment that can be found in deep biofilm layers in the lungs of CF patients [18]. How *B. cenocepacia* H111 is able to survive without oxygen is not understood; however, it was shown that the strain is unable to ferment arginine or reduce nitrate to nitrite or dinitrogen for energy production [18,22]. In this study, we applied Tn-seq to identify potential fitness determinants for the anoxic survival of *B. cenocepacia* H111. Nitrate was used in order to accompany any non-annotated nitrate reductases. Twenty-eight of the seventy-one identified genes were reported to be up-regulated under low oxygen conditions [22], and ten belong to the shared genetic determinants for the anoxic survival identified in this study, including *anr_2_*, *fliA* and genes encoding flagella components (i.e., *flhC*, *motB*, *flgG*, *flgF*). Among the genes required for anoxic survival in the absence of nitrate was the gene coding for the universal stress protein A (*uspA*, *I35_RS16815*), which is located upstream of *anr*_2_ (Figure 3) and was shown to be highly up-regulated in low oxygen conditions in *B. cenocepacia* J2315 [22]. Interestingly, the *lxa* locus of *B. cenocepacia* encodes six universal stress proteins that are almost exclusively up-regulated in response to low oxygen [22]. Furthermore, the analysis of sequential *B. cenocepacia* isolates from two adult male siblings with CF revealed that proteins encoded on the *lxa* locus, including the six USPs, are consistently up-regulated across both sets of patient isolates over time, suggesting that the *uspA* genes are important in vivo and likely to be important for niche adaptation to the hypoxic CF lung [36]. Some of the genes (*anr_2_, roxS/roxR* and *fliA)* identified in this study were also shown to be up-regulated in micro-oxic conditions (0.5% O_2_) using a combined transcriptomics and proteomics approach [18]. An *anr_2_* homolog was also identified in the study of Lieberman et al., who sequenced the genomes of 114 *Burkholderia dolosa* strains isolated from 14 CF patients over 16 years. The large number of mutations in the homolog and other oxygen-sensing regulators indicate that these genes are under selective pressure and are potentially important for chronic infection of *B. dolosa,* another *Bcc* member [37]. An *anr_2_* ortholog also exists in *B. thailandensis* E254 and was shown to be required for growth under denitrifying conditions [21]. Interestingly, although the *anr_2_* paralog *anr_1_* was not detected in our Tn-seq analysis, an *anr_1_* mutant showed impaired anaerobic survival relative to the wild-type (data not shown). Given that the *anr_2_ anr_1_* double mutant is less viable than the single mutants in the absence of oxygen, the two *anr* genes may be functionally redundant. The reason that only *anr_2_* provides a fitness benefit is currently unknown, and further investigations are required. In *B. dolosa*, the FixL/J two-component system is activated under low oxygen conditions. It was shown that the FixL senses the oxygen tension and activates the response regulator FixJ, which induces transcription of *fixK* [38], which is a homolog of *anr_2_*. Interestingly, *B. cenocepacia* H111 has genes coding for a homologous FixL/J system (*I35_RS10705*-*I35_RS10710*), which showed lower transposon insertion frequencies (but was below the threshold value for our Tn-seq fitness gene list) under anoxic conditions, suggesting a potential role of this two-component system in anaerobic survival. Many bacteria can adapt their metabolism to low oxygen tension by expressing terminal oxidases with high oxygen affinities [39,40]. Many bacteria express B- and C-class heme copper oxidases such as cytochrome *cbb*_3_ or *ba*_3_ oxidases as high (oxygen) affinity terminal oxidases [41,42,43,44]. However, these enzymes are absent in *Burkholderia* [45], raising the question of which oxidases are used under low oxygen tensions. Transcriptomic studies in *B. cenocepacia* found that the cytochrome *bd* oxidase is overexpressed under micro-oxic conditions [18,22], indicating that the cytochrome *bd* oxidase is used for survival at low oxygen tensions. Here, we identified the cytochrome *bd* oxidase genes *cydP*, *cydA* and *cydB* as fitness determinants for anoxic survival and a Δ*cydA* mutant showed significantly lowered survival under anoxic conditions. Together with the transcriptomic studies, our results highlight the importance of the *bd* oxidase for the adaption of *B. cenocepacia* to micro-oxic and anoxic conditions. Such conditions are often found in host-associated situations such as CF, and indeed, we could show that the Δ*cydA* mutant decreases the pathogenicity of *B. cenocepacia* H111 in *G. mellonella*. Since both the cytochrome *bd* oxidase cluster and the *anr_2_* gene were found to be involved in anoxic survival, we looked at potential binding boxes for Anr in the upstream region of the cytochrome *bd* oxidase cluster. Indeed, we were able to find a potential consensus sequence (TTGATCTCGATCAA) [46] in the promoter region, suggesting direct control of the cytochrome *bd* oxidase (I35_RS14430-14440) by Anr (Figure S3). Given the fact that Anr is known to positively regulate the *cbb*_3_ oxidase-2 of *P. aeruginosa* [41], which is overexpressed under low oxygen conditions, it is possible that Anr has a similar regulatory role in the regulation of cytochrome *bd* oxidase expression in *B. cenocepacia*. Further studies are needed to elucidate the role of cytochrome *bd* oxidase in the presence of nitrate. We performed a nitrate reduction test, which showed that nitrate is still present after five days of anoxic incubation, and no nitrite was produced (data not shown), further corroborating the fact that *B. cenocepacia* H111 is unable to reduce nitrate to nitrite. Another regulatory system identified in this study is the two-component system RoxS/RoxR, which is homologous to RoxS/RoxR of *B. thailandensis* E264 [21] and *P. aeruginosa* PAO1 [29]. In *B. thailandensis,* the *roxS* mutant was unable to grow in the presence of nitrite under denitrifying conditions and showed an accumulation of N_2_O, indicating an involvement of this two-component system in the regulation of nitrite reduction [21]. The RoxS/RoxR system in *P. aeruginosa* positively regulates the expression of the cyanide insensitive oxidase genes [20]. The cytochrome *bd* oxidase cluster identified in our studies (*I35_RS14430-14440*) possesses a potential RoxR consensus sequence (GCGGCAATTTAGAGC) [47] in the promoter region (Figure S3). This suggests that in addition to Anr, RoxS/RoxR may directly control the expression of the cytochrome *bd* oxidase in *B. cenocepacia* H111. In *B. pseudomallei,* the RegA/RegB (homologous to RoxS/RoxR) is essential for anoxic growth in the presence of nitrate. Additionally, it was shown that this two-component system is also required for full *B. pseudomallei* virulence in the murine infection model [48]. In line with this observation, we observed reduced virulence of the Δ*roxS* mutant in *B. cenocepacia* H111 using a *G. mellonella* infection model, suggesting that this two-component system is controlling the virulence of *B. cenocepacia* H111. Bernier et al. showed that the RoxS/RoxR system is also involved in the resistance of *B. cenocepacia* K56-2 towards phenazines produced by *P. aeruginosa* [49]. The identification of flagella biosynthesis sigma factor *fliA* as a fitness determinant for anoxic survival may be due to the importance of flagella in swimming toward the surface where the possibility to encounter oxygen is higher. Despite the fact that the FliA regulator of *P. aeruginosa* plays a role in virulence by influencing colonization ability and hemolytic activity, the absence of the FliA regulator did not affect *B. cenocepacia* H111 virulence in *G. mellonella* [35]. During our experimental work, we observed an induction of swarming when *B. cenocepacia* H111 wild-type was exposed to micro-oxia, and this induction was independent from the regulators we investigated in this study (Anr, RoxR/S, FliA). We further observed that nitrate inhibits swarming under aerobic conditions and in micro-oxic conditions. However, nitrate has no negative or positive effect on the swimming ability of *B. cenocepacia* H111 in aerobic and micro-oxic conditions. In *P. aeruginosa,* nitrate has no effect on swarming [50]. We also identified the flagellar master operon *flhDC* to be important for anoxic survival. Studies in *E. coli* K-12 showed that loss of FlhD altered not only the expression of genes involved in flagella biosynthesis but also influenced anoxic respiration by regulating the expression of the oxygen sensor Aer, the *nap* gene cluster (periplasmatic nitrate reductase) and a cluster encoding for a nitrite reductase (*nrfABCDE*). Because FlhD/FlhC also negatively regulates the genes involved in aerobic respiration, such as cytochrome *bo_3_* ubiquinol oxidase (*cyoBCDE*) or succinate dehydrogenase (*sdhCDAB*), the FlhD/FlhC complex may play a role as a switch in the transition of aerobic to anoxic respiration [51]. An important survival mechanism of *P. aeruginosa* under anoxic conditions is the production of excreted phenazines allowing bacteria to generate ATP by reducing phenazines and re-oxidizing them far away by oxygen or other oxidants [12]. Some strains of the *Bcc,* such as *Burkholderia lata* ATCC 17760, contain the core phenazine biosynthesis genes encoded in a conserved seven gene operon (*phzABCDEFG*) [52], and phenazine production has been reported for *B. cepacia* strain 5.5B and *B. cenocepacia* strain K56-2 [53,54]. *B. cepacia* is also known to produce other redox-active compounds, such as pyrrolnitrin [55], which might have similar functions as phenazines. Therefore, it is possible that *B. cenocepacia* H111 might employ phenazine or phenazine-like compounds for survival under anoxic conditions, and this is an interesting question for future studies. 

## 4. Materials and Methods

### 4.1. Bacterial Strains, Media and Growth Conditions

Bacterial strains, oligonucleotides and plasmids used throughout this study are listed in Table S1. Bacterial cultures for routine culture were grown either in lysogeny broth (LB) [56] or AB medium [57] supplemented with 10 mM sodium citrate (ABC) at 37 °C shaking at 220 rpm for aerobic (21% O_2_) conditions, at 37 °C statically for anoxic conditions (<1.0% O_2_, GasPak EZ Anaerobe Container System (BD, Becton, Dickinson and Company, Sparks, NV, USA, Ref: 260678) or micro-oxic conditions (6–16% O_2_, BD GasPak EZ container system). To maintain micro-oxic conditions, the micro-oxic sachet (GasPak Ref: 260680) was changed every 24 h. For the Tn*23 B. cenocepacia* H111 library [24], 0.2% l-rhamnose was added to the medium at the beginning of the experiment. If required, appropriate antibiotics were supplemented using the following concentrations given in micrograms per milliliter for *B. cenocepacia* H111 [58] chloramphenicol (Cm) 80, kanamycin (Km) 50, gentamycin (Gm) 20, trimethoprim (Tm) 100 and for *Escherichia coli* Km 50, Cm 20, Tm 50, Gm 10. 

### 4.2. Tn-seq Technology

The Tn*5*-based transposon library of *B. cenocepacia* H111 used in this study contains approximately 1,000,000 unique insertion mutants and was previously constructed and used by our group [24]. A washed preculture of the *B. cenocepacia* H111 Tn*23* library was inoculated in ABC (OD_600_ of 0.02, corresponding to roughly 10^7^ CFUs per ml) supplemented with 0.2% l-rhamnose and left to grow until the end of the exponential phase (OD_600_ = 0.8). This preculture was then used to inoculate a 100 mL flask containing 25 mL ABC with 0.2% l-rhamnose to a starting OD_600_ of 0.02, and the culture was grown until mid-exponential phase (OD_600_ = 0.5) (control sample, approximately 5 generations). For the test samples (ABC or ABC with 10 mM NaNO_3,_ both amended with 0.2% l-rhamnose), 6 times 6 mL in 14 mL capped glass tubes were inoculated with the same preculture to the same starting OD_600_ as the control sample. The tubes were anoxically incubated in a jar for 5 days. Thereafter, the six tubes for each condition were combined in a 100 mL sterile flask and grown until OD_600_ 0.5 under aerobic conditions (220 rpm, 37 °C). The cells were then pelleted and stored at −20 °C. The genomic DNA from the control and the test samples was extracted using the GeneElute^TM^ Bacterial Genomic DNA kit (Sigma-Aldrich, St. Louis, MO, USA, NA2110) and prepared for Illumina sequencing using the circle method [59], as described by [21]. In brief, the genomic DNA was sheared by using Covaris (E220 focused ultrasonicator, Covaris^TM^, MA, USA) into 300 bp long fragments. The NEBNext Ultra II DNA Library Prep Kit (New England Biolabs (NEB), Ipswich, MA, USA, E7645S) was used for end repair. Adaptors were ligated, followed by a restriction digestion at the *BamH*I site within the transposon. Circularization followed by exonuclease treatment with *Exo*I (NEB, M0293L), T7 Gene 6 *Exo* (NEB, M0263S) and Lambda *Exo* (NEB, M0262S) occurred with size selected DNA fragments. DNA was sequenced (Miseq, Ilumina, San Diego, CA, USA) by using the paired end (2 times 150 bp) Miseq Reagent kit V2 using 300 cycles (Ilumina, San Diego, CA, USA, MS-102-2002). Analysis of the sequenced DNA libraries and a fitness analysis was performed as previously described [21]. In brief, the open-source software Tn-seq Explorer [60] was used to calculate the unique insertion density (UID), which is the unique insertion counts (UIC) of the transposon in a gene divided by its gene length in base pairs to normalize for gene sizes. Thereafter, the UID was normalized (nUID) by dividing the UID by the total UIC per sample (Table 1). After the fitness analysis, a list of potential fitness determinants was obtained by using the following parameters: log_2_ fold change (FC) of at least −1 (FC = nUID of the test sample divided by the nUID of the control sample) and a difference in nUID between the control and the test samples of at least 0.009. Those parameters are strict but ensure a restricted list of fitness determinants with increased likelihood to show a strong fitness effect in anoxic conditions. Clusters of Orthologous Groups (COG) were added to the fitness list by using the online tool EggNog version 4.5.1 [25]. The heatmap was created in Rstudio (PBC, Boston, MA, USA) version 4.0.3 using the following packages: readxl and pheatmap with the command cluster_rows = TRUE. 

### 4.3. Construction of B. cenocepacia H111 Mutant Strains

To validate the Tn-seq data, three deletion mutants of gene *anr_2_* (*I35_RS16810*), *cydA* (*I35_14435*) and *roxS* (*I35_RS15825*) were generated according to the deletion mutagenesis system established by Flannagan and colleagues [61]. The genomic DNA of *B. cenocepacia* H111 wild-type as PCR template was extracted by using the GenElute^TM^ Bacterial Genomic DNA Kit. PCRs for the up- and downstream regions were carried out by using HF phusion polymerase (Sigma, St. Louis, MO, USA, F-530L). The following primer pairs were used for the upstream flanking site amplification of the above mentioned genes: anr_2_up_Fw_KpnI and anr_2_up_Rv_XbaI (size: 654 bp), cydA_Rv_up_NdeI and cydA_Fw_up_XbaI (size: 647 bp), roxSup_Fw_KpnI and roxSup_Rv_XbaI (size: 581 bp). For the downstream regions the primers anr_2_dw_Rv_KpnI and anr_2_dw_Fw_EcoRI (size: 574 bp), cydA_Fw_dw_NdeI and cydA_Rv_dw_EcorRI (size: 615 bp), roxSdw_Fw_EcoRI and roxSdw_Rv_KpnI (size: 505 bp) were used. The upstream and downstream fragments were ligated into the suicide plasmid, pGPI-SceI, which was previously extracted by using the following kit: QIAprep Spin Miniprep Kit (Qiagen, Hilden, Germany, Ref: 27106). The ligation mixture was transformed into competent *E. coli* SY327 λpir cells, and the transformants were plated on selective plates. The plasmids (pGPI-SceI_anr_2_updw, pGPI-SceI_cydAupdw, pGPI-SceI_roxSupdw) carrying up- and downstream regions were integrated into *B. cenocepacia* H111 wild-type via triparental mating (TPM). Double strand break at the I-SceI endonuclease site was initiated by introducing the plasmid pDAI-SceI *E. coli* pDAI-SceI S17λpir via biparental mating with the transconjugants. The double mutant of the *anr* genes was made in three steps. First, a deletion mutant of *anr_1_* (*I35_RS23120*) was constructed by using the Gateway Technology (Invitrogen, Waltham, MA, USA). In brief, the 5′ and 3′ regions of *anr_1_* were amplified by using primers pairs: anr_1_up_F and anr_1_up_R, and anr_1_dw_F and anr_1_dw_R. The kanamycin resistance cassette (KRC) was amplified from the plasmid pKD4 with the primers pKD4rev-2 and pKD4fwd-2. These three PCR products were annealed by using Ex Taq Polymerase (Takara Bio, San Jose, CA, USA), and then amplified with the primer set GWattB1 and GWattB2 by PCR. The resultant fragment consisting of 5′ region, KRC and 3′ region was cloned into pDONR221 using the BP clonase II kit (Invitrogen, Thermo Fisher Scientific, Waltham, MA, USA), which was then transformed into competent *E. coli* Top 10 cells. LR clonase kit II (Invitrogen, Thermo Fisher Scientific, MA, USA) was used to genetically transfer the insert from pDONR221 into the suicide vector pAUC40, which was transformed into *E. coli* cc118λpir. The mutagenesis targeting pAUC40_anr_1_updw plasmid was transferred into *B. cenocepacia* H111 by TPM to generate the *anr1* deletion mutant. To mutate *anr_2_* (*I35_RS16810*), a 259 bp region of *anr*_2_ was first amplified by using primers anr_2__IM_F and anr_2__IM_R, which was subsequently ligated directly into pGEM T easy vector (Promega, Madison, WI, USA). The fragment was released by digestion with *Xho*I and *Bgl*II and then subcloned into cut pSHAFT2 [21] to generate the *anr_2_* insertional mutagenesis construct. Plasmid pSHAFT2_anr_2_ was conjugated into the wild-type and the deletion mutant *anr_1_* by TPM. The *fliA* (*I35_RS00760*) mutant was created by insertional mutagenesis using the suicide plasmid pSHAFT2. An internal part of *fliA* was amplified with the following primers fliA_Fw_EcoRI and fliA_Rv_XbaI (size: 238 bp), ligated into pSHAFT2 and later transformed into *E. coli* cc118λpir. The correct plasmid was called pSHAFT2_*fliA* and brought into the *B. cenocepacia* H111 wild-type genome by TPM. Complementation of the *fliA* was obtained by amplifying the entire gene using the primers fliA_comp_Rv_XbaI and fliA_comp_Fw_EcoRI (size: 749 bp) and inserting it into the plasmid pBBR1MSC-5. The plasmid pBBR1MSC-5_*fliA* with the entire gene was transferred into the *fliA* mutant via TPM resulting in the complemented strains *fliA*^+^.

### 4.4. Phenotypical Analyses

For the anoxic survival experiment, bacterial cultures were adjusted to a starting OD_600_ of 0.02 in ABC or ABC supplemented with 10 mM NaNO_3_ in 14 mL capped glass tubes to a final volume of 6 mL (approximately 2.5 × 10^7^ cells per mL). The adjusted cultures were incubated at 37 °C statically in anoxic condition for 5 days as described above. Colony forming units were determined on LB plates by serial dilutions in 0.9% NaCl before and after 5 days of anoxic incubation. The normalized survival percentage relative to the wild-type was calculated by dividing the CFU pro mL after the anoxic experiment for each tested strain by the average of CFU pro ml at the beginning of the inoculum, multiplied by 100. Swarming and swimming were performed as previously described [18,62]. In brief, adjusted cultures (OD_600_ = 0.5) were spotted onto 0.4% or 0.2% ABC or ABC with 10 mM NaNO_3_ agar plates supplemented with 0.1% Bacto^TM^ casamino acids (BD, Waltham, MA, USA) and incubated at 37 °C for three days or 24 h either in aerobic or in micro-oxic conditions. For the Transmission Electron Microscopy (TEM), the overnight culture of bacteria was ten-fold diluted in saline solution. The 20 µL of bacterial suspension was placed on a glow-discharged formvar-coated 300-mesh copper grid (Plano GmbH, Wetzlar, Germany). After 1 min, the excess liquid was removed using filter paper. The grid was negatively stained with 1% uranyl acetate for 1 min and dried with filter paper in a glass petri dish for 30 min. The sample was visualized using an FEI Tecnai G2 Spirit TEM (FEI, Hillsboro, OR, USA) at 120 kV acceleration voltage with the detector side-mounted digital camera Gatan Orius 1000 (4 k × 2.6 k pixels). Biofilm formation was performed as previously reported [58] in ABC medium or in ABC amended with 10 mM NaNO_3_. Wild-type and mutants were stained with crystal violet for 30 min. After washing the 96-well plates with deionized water, the attached crystal violet was solubilized by the addition of 120 µL of DMSO (Sigma, St. Louis, MO, USA) and left for 20 min at room temperature (RT). Biofilm formation was measured by a plate reader (TECAN, Infinite M200 pro, tecan biotechnology and life science, Taunton, MA, USA), which was measured at 570 nm. 

### 4.5. Pathogenicity Experiments Using Galleria mellonella

*G. mellonella* larvae were purchased from BioSystems Technology (TruLarv, Exeter, UK) or the fishershop in Zurich (Reptile-Food.ch) and used immediately after arrival or kept at 16 °C for a maximum of 2 days. The infection assay was carried out as described previously with the following modifications [33]. The cultures were diluted to an OD_600_ of 0.0625 (± 2–3 × 10^7^ CFU per mL) in 10 mM MgSO_4_, and thereafter, 10 µL were injected into *G. mellonella* (approximately 200,000 cells) with a 27 gauge needle (BD Microlance, Waltham, MA, USA, Ref: 302,200). Three or more biological replicates were performed with 10 larvae for each sample. The statuses of the larvae were checked to be alive (responsive) or dead (non-responsive) every 24 h.

### 4.6. Statistical Analyses 

GraphPad prism version 5.01 (San Diego, CA, USA) was used to analyze the data obtained during this study. One-way ANOVA was used (Dunnett) with a confidence interval of 99%. For Biofilm formation, unpaired *t*-test was used.

## Figures and Tables

**Table 1:** Number of *B. cenocepacia* H111 unique insertion counts (UIC) and potential fitness determinants identified in each tested condition. NA = not applied.

Condition	Medium	Number of UIC	Number of Potential Fitness Determinants
Aerobic	ABC	1,106,798	NA
Anoxic	ABC	987,541	61
Anoxic	ABC + 10 mM NaNO_3_	841,867	30

**Table 2:** List of the 20 identified common fitness determinants for anoxic survival of *B. cenocepacia* H111.

Locus_tag	Orthologs ^1^	Description	Gene Name ^4^	COG ^2^	FC ^3^ (ABC)	FC ^3^ (ABC + 10 mM NaNO_3_)
*I35_RS00665*	*BCAL0125*	transcriptional regulator	*flhC*	K	−1.27	−1.02
*I35_RS00675*	*BCAL0127*	flagellar motor protein	*motB*	N	−2.32	−1.01
*I35_RS00760*	*BCAL0144*	RNA polymerase sigma factor	*fliA*	K	−1.71	−1.33
*I35_RS01950*	*BCAL0387*	GTP-binding protein	*-*	J	−1.7	−1.33
*I35_RS02485*	*BCAL3398*	competence damage-inducible protein A	*-*	S	−2.19	−1.35
*I35_RS02685*	*BCAL3358*	ABC transporter	*gltI*	P	−1.43	−1.12
*I35_RS02700*	*BCAL3354*	arginine ABC transporter ATP-binding protein	*-*	E	−1.88	−1.04
*I35_RS03285*	*-*	tRNA-Lys	*-*	-	−1.03	−1.28
*I35_RS04855*	*-*	nicotinate phosphoribosyltransferase	*-*	H	−4.48	−2.37
*I35_RS06995*	*BCAL1509*	tRNA pseudouridine synthase B	*truB*	J	−1.9	−1.6
*I35_RS07080*	*BCAL1526*	pilus assembly protein	*tadE*	U	−2.41	−1.52
*I35_RS13950*	*BCAL0883*	TetR family transcriptional regulator	*-*	K	−1.22	−1.45
*I35_RS14265*	*BCAL0820*	adenine phosphoribosyltransferase	*-*	F	−2.01	−2.02
*I35_RS15445*	*BCAL0576*	flagellar hook protein	*flgK*	N	−2.26	−1.18
*I35_RS15470*	*BCAL0569*	flagellar basal body rod protein	*flgG*	N	−2.23	−1.35
*I35_RS15820*	*BCAL0499*	chemotaxis protein	*roxR*	T	−3.26	−3.17
*I35_RS15825*	*BCAL0497*	two-component system sensor histidine kinase	*roxS*	T	−2.08	−2.43
*I35_RS15950*	*BCAL0473*	7-cyano-7-deazaguanine synthase	*-*	S	−1.14	−1.1
*I35_RS16810*	*BCAM0049*	Crp-Fnr family transcriptional regulator	*anr_2_*	K	−1.32	−2.86
*I35_RS28070*	*BCAM2434*	YsiA protein	*-*	K	−2.84	−1.98

^1^ Name of *B. cenocepacia* J2315 ortholog [27]. ^2^ Cluster of orthologous groups (COG), amino acid transport and metabolism [E], nucleotide transport and metabolism [F], coenzyme transport and metabolism [H], translation, ribosomal structure and biogenesis [J], transcription [K], cell motility [N], inorganic ion transport and metabolism [P], function unknown [S], signal transduction mechanisms [T], intracellular trafficking, secretion and vesicular transport [U] (https://www.ncbi.nlm.nih.gov/research/cog/ accessed on 8 October 2021). ^3^ The log_2_ fold change (FC) of the normalized UID (nUID) of the control sample (ABC aerobic) and the test sample (ABC or ABC with 10 mM NaNO_3_ in anoxic condition). ^4^ Gene name from *B. cenocepacia* J2315 [27] or through homology search (*anr* and *rox* system).

## Data Availability

Raw FASTQ files generated from the Illumina MiSeq platform are publicly available from the NCBI short reads archive (SRA) and can be found under accession no: PRJNA823902. Each sample can be found under the following accession numbers: aerobic sample in ABC, SAMN27365712; anoxic sample in ABC and ABC with NO_3_ SAMN27365713.

## Supplementary Materials

The following supporting information can be downloaded at: https://www.mdpi.com/article/10.3390/ijms23094560/s1. Refs. [21,58,59,61,63,64,65,66,67,68,69,70,71,72,73,74] are cited in the Supplementary Material.
