# Supplementary material for: Identification of Key Factors for Anoxic Survival of B. cenocepacia H111
Source: Int J Mol Sci. 2022 Apr 20;23(9):4560. doi: 10.3390/ijms23094560 (PMC9104464; doi:10.3390/ijms23094560)
Supplement: Supplementary file 1 [file ijms-23-04560-s001.zip › ijms-1648933-Supplementary Material Explanation.pdf]

**Supplementary Material**

Figure S1. Genetic determinants for each condition were classified into COGs. X-axis = COG categories, y-axis = percentage of each COG represented in an anaerobic condition (color indicates specific anaerobic condition). COG categories description: C =energy production and conversion, E = amino acid transport and metabolism, F = nucleotide transport and metabolism, G =carbohydrate transport and metabolism, H = coenzyme transport and metabolism, I = lipid transport and metabolism, J = translation, ribosomal structure and biogenesis, K = transcription, L = replication, recombination and repair, M = cell wall, membrane and envelope biogenesis, N = cell motility, O = post-translational modification, protein turnover, and chaperones P = inorganic ion transport and metabolism, S = function unknown, T = signal transduction mechanisms, U = intracellular trafficking, secretion, and vesicular transport, V = defense mechanisms, Na = not applicable. The percentage obtained by dividing the COG number found within a condition by the total number of this specific COG category found in *B. cenocepacia* H111 among all genes multiplied by 100. The asterisks represent statistical significance (Fisher test, *p*-value *** < 0.0001, ** < 0.01).; Figure S2: Biofilm formation in *B. cenocepacia* H111 wild-type, *fliA* deletion mutant and *fliA*$^+$ complemented strain at 30 °C after 48 hours in ABC **A)** and ABC with 10 mM $NaNO_3$ **B)**. X-axis = strain, y-axis = $OD_{570}$ in nm. T-test, n=6, error bars=SD, p-value < 0.05 = *, p-value < 0.0001 = ***.; Figure S3: Upstream promoter region of the *cydPAB* cluster (*I35_RS1443-14440*) in *B. cenocepacia* H111. Shown is the + strand and the -10/-35 box as well as the start codon of the *cydP* unit of the cluster. Potential Anr and RoxR binding sites are indicated in red and green, respectively. Table S1: List of strains, oligonucleotides and plasmids used in this study.; Table S2: Table of the 61 fitness determinants found in ABC in anoxia and Table S3 list of 30 fitness determinants found in ABC with nitrate in anoxia.
