# Supplementary material for: Identification of Key Factors for Anoxic Survival of B. cenocepacia H111
Source: Int J Mol Sci. 2022 Apr 20;23(9):4560. doi: 10.3390/ijms23094560 (PMC9104464; doi:10.3390/ijms23094560)
Supplement: Supplementary file 1 [file ijms-23-04560-s001.zip › S1 Table Strains, oligos, plasmids.pdf]

Table S1. List of strains, oligonucleotides and plasmids used in this study

Strain or plasmid	Description	Reference
Strains		
E. coli		
cc118λ-pir	Δ(ara-leu) *araD* Δ*lacX74 galE galK phoA20 thi1 rpsE rpoB argE(Am) recAl* λ pir; $Strep^R$	[63]
Top10	Δ*lacX74 ara*Δ*139*Δ*(ara-leu)*	Invitrogen
SY327 λpir	Δ*araD*, (lac pro) argE(Am) recA56 rif^R nalA λpir	[64]
B. cenocepacia		
wild-type	CF isolate from Germany, genomovar III	[65,58]
Δ*roxS*	Two-component family of histidine kinase Δ*roxS (I35_RS15825)*	This study
Δanr_2	Crp/Fnr family transcriptional regulator Δanr_2 *(I35_RS16810)*	This study
Δanr_1	Crp/Fnr family transcriptional regulator Δanr_1 *(I35_RS23120)*	This study
Δ*cyto*	Cytochrome BD oxidase subunit I Δ*cyto (I35_RS14435)*	This study
Δanr_1-anr_2	Δanr_1 (*I35_RS23120*) deletion and anr_2 (pSHAFT2*::I35_RS16810)* double mutant, Km^R , Cm^R	This study
fliA	*fliA* (I35_RS00760) insertional mutant in pSHFAT2	This study
$fliA^+$	fliA complemented strain with pBBRMCS-5	This study
P. aeruginosa		
PA14	Wild type, UCBPP-PA14, human isolate	[66]
Plasmids		
pRK2013	Helper plasmid; Km^R	[67]
pSHAFT2	Broad-host-range suicide plasmid, mobilisable for conjugation; Cm^R	[68]
pBBR1MCS-5	Broad host-range cloning vector; Gm^R	[69]
pDAI-SceI	Suicide plasmid with oriR6K, mob+ , I-SceI restriction site, Tm^R	[61]
pDONR221	Km^R, Cm^R, attP sites, ccdB, pUC origin	Invitrogen
pGPI-SceI	Suicide plasmid with oriR6K, mob+ , I-SceI restriction site, Tm^R	Steiner unpublished, [61]
pAUC40	Cm^R , $Strep^R$ attR sites, R6K origin, sacB, ccdB	[70]
pBBR5::FLP	Gm^R, sacB	[71]
pKD4	Km^R	[72]
pGEM T easy vector	PCR cloning	Promega
pGPI-SceI_anr_2updw	pGPI-SceI containing the upstream (654 bp) and downstream (560 bp) regions of the anr_2 (*I35_RS16810*), Tm^R	This study
pGPI-SceI_cydAupdw	pGPI-SceI containing the upstream (647 bp) and downstream (615 bp) regions of the *cydA* (*I35_RS14435*), Tm^R	This study
pGPI-SceI_roxSupdw	pGPI-SceI containing the upstream (581 bp) and downstream (505 bp) regions of the *roxS* (*I35_RS15825*),Tm^R	This study
pAUC40_anr_1updw	pAUC40 containing upstream (466 bp) and downstream (495 bp) regions of anr_1 (*I35_RS23120*), Cm^R	This study

pSHAFT2_anr$_2$	pSHAFT2 containing a 259 bp region of *anr$_2$* (I35_RS16810), Cm^R	This study
pSHAFT2_*fliA*	pSHAFT2 containing a 238 bp region of *fliA* (*I35_RS00760*), Cm^R	This study
pBBR1MSC-5_*fliA*	pBBR1MSC-5 containing the entire *fliA* gene (*I35_RS00760*) (749 bp), Gm^R	This study
<u>Oligonucleotides</u>		
roxSup_Fw_KpnI_	tccggtaccCCGAGCAGTTTACCCGTTTA	This study
roxSup_Rv_XbaI	tcctctagaAACAGGCCGAATTGTACAGG	This study
roxSdw_Fw_EcoRI	tccgaattcGTCGCCGAGATGTTGTTGTT	This study
roxSdw_Rv_KpnI	tccggtaccGGCCTCAACCAACACGTAGA	This study
anr$_2$up_Fw_KpnI	tccggtaccGATCGTATACTCCGGCGTTG	This study
anr$_2$up_Rv_XbaI	tcctctagaTACTCTGCCTCGCAGCATC	This study
anr$_2$dw_Rv_KpnI	tccggtaccTGCAACACCTCTGATACCGC	This study
anr$_2$dw_Fw_EcoRI	tccgaattcTCGACGATCAGGATTACACG	This study
cydA_Rv_up_NdeI	tcccatatgGCTACTTATCATGGTGGTCT	This study
cydA_Fw_up_XbaI	tcctctagaCGA ATA CGC GTA AAT CGC G	This study
cydA_Fw_dw_NdeI	tcccatatgTGAGTACGCCCTCGCAAC	This study
cydA_Rv_dw_EcoRI	tccgaattcACG CCG TCG GTC TTC ATC	This study
fliA_Fw_EcoRI	tccgaattcGACGAATACCAGGGGATGC	This study
fliA_Rv_XbaI	tcctctagaGCAGATTCAGACCCCGTTC	This study
fliA_comp_Rv_XbaI	tcctctagaTCGACAAGGGGTCAGGACT	This study
fliA_comp_Fw_EcoRI	tccgaattcGCCATGATGTACAACGCTCA	This study
pSHAFT_Fw	CTTCAGCTGATGTGTGATAACATACT	[73]
anr$_2$_IM_F	GCCTCGAGTCGGTCATCTG	This study
anr$_2$_IM_R	agatctCTGTACGGAATCACGCAGAC	This study
anr$_2$_Comp_F	tccaagcttGGTGGGACAATGATTTCGTC	This study
anr$_2$_Comp_R	tcctctagaGTGCGGTATCAGAGGTGTTG	This study
anr$_1$_up_F	CAATTTCGGCGGCGCAACAC	This study
anr$_1$_up_R	TGGCGGTGGACATGAGTCG	This study
anr$_1$_dw_F	ATCATCGACGCGCAAGCGCT	This study
anr$_1$_dw_R	AAACACGTGCCGCCTCCATC	This study
pKD4fwd-2	TAGGCTGGAGCTGCTTCGAAGTTC	This study
pKD4rev-2	CATATGAATATCCTCCTTAGTTCCTATTCCG	This study
GWattB1	GGGGACAAGTTTGTACAAAAAAGCAGGCT	[74]
GWattB2	GGGGACCACTTTGTACAAGAAAGCTGGGT	[74]
PAIR_ADAPT_6BC##_HI	ACACTCTTTCCCTACACGACGCTCTTCCGATCTBBBBBBT	[59]
PAIR_ADAPT_6BC##_LO_5PH	BBBBBBAGATCGGAAGAGCGGTTCAGCAGGAATGCCGAG	[59]
T23_PAIR_COLLECT	CTTCGGCGCGCCCTAGGGGGATCCTCGGCATTCCTGCTGAACCGCTCTTCCGATCT	[59]
T23_SLXA_PAIR_Amp	GGAACTTCGGAATAGGAACTTCTTAGATGTGTATAAGA G	[59]
SLXA_PAIR_REV_AMP	CAAGCAGAAGACGGCATACGAGATCGGTCTCGGCATTC CTGCTGAACCGCTCTTCCGATCT	[59]
T23_SEQ_G (Fw)	ATTAGGAACTTCGGAATAGGAACTTCTTAGATGTGTATAAGAGACAG	[21]
T23_INDEX_1 (Index)	CTAGAGAATAGGAACTTCGGAATAGGAACTTCTTAGATGTGTATAAG	[59]
PE_READ2_SEQ (Rv)	CGGTCTCGGCATTCCTGCTGAACCGCTCTTCCGATCT	[59]

Reference

21. Vitale, A.; Paszti, S.; Takahashi, K.; Toyofuku, M.; Pessi, G.; Eberl, L. Mapping of the denitrification pathway in *Burkholderia thailandensis* by genome-wide mutant profiling. *J. Bacteriol.* **2020**, *202*. e00304-20. https://doi.org/10.1128/JB.00304-20.
58. Gotschlich, A.; Huber, B.; Geisenberger, O.; Togl, A.; Steidle, A.; Riedel, K.; Hill, P.; Tümmler, B.; Vandamme, P.; Middleton, B.; et al. Synthesis of multiple N-acylhomoserine lactones is wide-spread among the members of the *Burkholderia cepacia* complex. *Syst. Appl. Microbiol.* **2001**, *24*, 1–14. https://doi.org/10.1078/0723-2020-00013.
59. Gallagher, L.A.; Ramage, E.; Patrapuvich, R.; Weiss, E.; Brittnacher, M.; Manoil, C. Sequence-defined transposon mutant library of *Burkholderia thailandensis*. *MBio* **2013**, *4*, 1–7. https://doi.org/10.1128/mBio.00604-13.
61. Flannagan, R, Linn, T, V.M. A system for the construction of targeted unmarked gene deletions in the genus *Burkholderia*. *Environ. Microbiol.* **2008**, *10*, 1652–1660. https://doi.org/10.1111/j.1462-2920.2008.01576.x.
63. Herrero, M., V de Lorenzo, Timmis, K. Transposon vectors containing non-antibiotic resistance selection markers for cloning and stable chromosomal insertion of foreign genes in Gram-negative bacteria. *J Bacteriol*, **1990**, *172*, 6557-6567. https://doi.org/10.1128/jb.172.11.6557-6567.1990.
64. Miller, V.L., Mekalanos, J.J. A novel suicide vector and its use in construction of insertion mutations: osmoregulation of outer membrane proteins and virulence determinants in Vibrio cholerae requires toxR. *Journal of bacteriology*, **1998,** *170*(6), pp.2575-2583. https://doi.org/10.1128/jb.170.6.2575-2583.1988.
65. Römling, U., Wingender, J., Müller, H. and Tümmler, B. A major Pseudomonas aeruginosa clone common to patients and aquatic habitats. *Applied and Environmental Microbiology*, **1994,** *60*(6), pp.1734-1738. https://doi.org/10.1128/aem.60.6.1734-1738.1994.
66. Rahme, L.G., Stevens, E.J., Wolfort, S.F., Shao, J., Tompkins, R.G. and Ausubel, F.M. Common virulence factors for bacterial pathogenicity in plants and animals. *Science*, **1995**, *268*(5219), pp.1899-1902. https://doi.org/10.1126/science.7604262.
67. Phadnis, S.H., Berg, D.E. Identification of base pairs in the outside end of insertion sequence IS50 that are needed for IS50 and Tn5 transposition. *Proceedings of the National Academy of Sciences*, **1987**, *84*(24), pp.9118-9122. https://doi.org/10.1073/pnas.84.24.9118.
68. Shastri, S., Spiewak, H.L., Sofoluwe, A., Eidsvaag, V.A., Asghar, A.H., Pereira, T., Bull, E.H., Butt, A.T., Thomas, M.S., . An efficient system for the generation of marked genetic mutants in members of the genus Burkholderia. *Plasmid*, **2017,** *89*, pp.49-56. https://doi.org/10.1016/j.plasmid.2016.11.002.
69. Kovach, M.E., Elzer, P.H., Hill, D.S., Robertson, G.T., Farris, M.A., Roop II, R.M., Peterson, K.M. Four new derivatives of the broad-host-range cloning vector pBBR1MCS, carrying different antibiotic-resistance cassettes. *Gene* **1995,** *166*(1), pp.175-176. https://doi.org/10.1016/0378-1119(95)00584-1.
70. Carlier, A., Burbank, L. and von Bodman, S.B., Identification and characterization of three novel EsaI/EsaR quorum-sensing controlled stewartan exopolysaccharide biosynthetic genes in Pantoea stewartii ssp. stewartii. *Molecular microbiology*, **2009**, *74*(4), pp.903-913.
71. Agnoli, K., Schwager, S., Uehlinger, S., Vergunst, A., Viteri, D. F., Nguyen, D. T., Sokol, P.A., Carlier, A., Eberl, L. Exposing the third chromosome of Burkholderia cepacia complex strains as a virulence plasmid. Molecular microbiology, **2012**, *83*(2), 362-378. http://doi.org/10.1111/j.1365-2958.2011.07937.x.
72. Wanner, B. L., Datsenko, K. A. One-step inactivation of chromosomal genes in Escherichia coli K-12 using PCR products. P Natl Acad Sci Usa, **2000**, *97*(12), 6640-6645. http://doi.org/10.1073/pnas.120163297.
73. Lardi, M., Liu, Y., Purtschert, G., Bolzan de Campos, S., Pessi, G. Transcriptome analysis of Paraburkholderia phymatum under nitrogen starvation and during symbiosis with Phaseolus vulgaris. Genes, **2017**, *8*(12), 389. http://doi.org/10.3390/genes8120389
74. Choi, K. H., Schweizer, H. P. An improved method for rapid generation of unmarked Pseudomonas aeruginosa deletion mutants. BMC microbiology, **2005**, *5*(1), 1-11. http://doi.org/10.1186/1471-2180-5-30.
