# Supplementary figures and images for: Identification of Key Factors for Anoxic Survival of B. cenocepacia H111
Source: Int J Mol Sci. 2022 Apr 20;23(9):4560. doi: 10.3390/ijms23094560 (PMC9104464; doi:10.3390/ijms23094560)

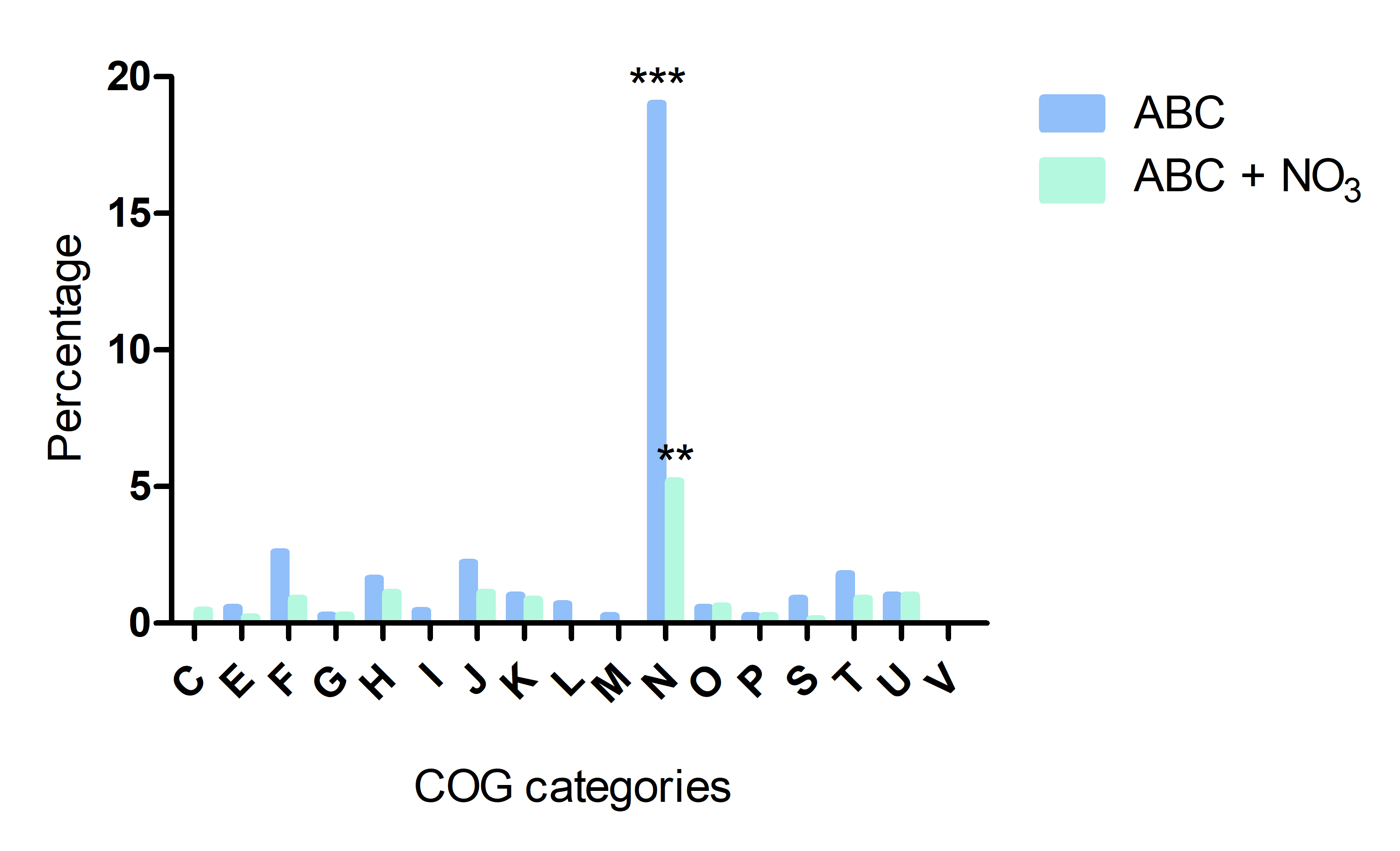

Supplement: Supplementary file 1 [file ijms-23-04560-s001.zip › Paszti et al_Supplemental Figure S1.tif]

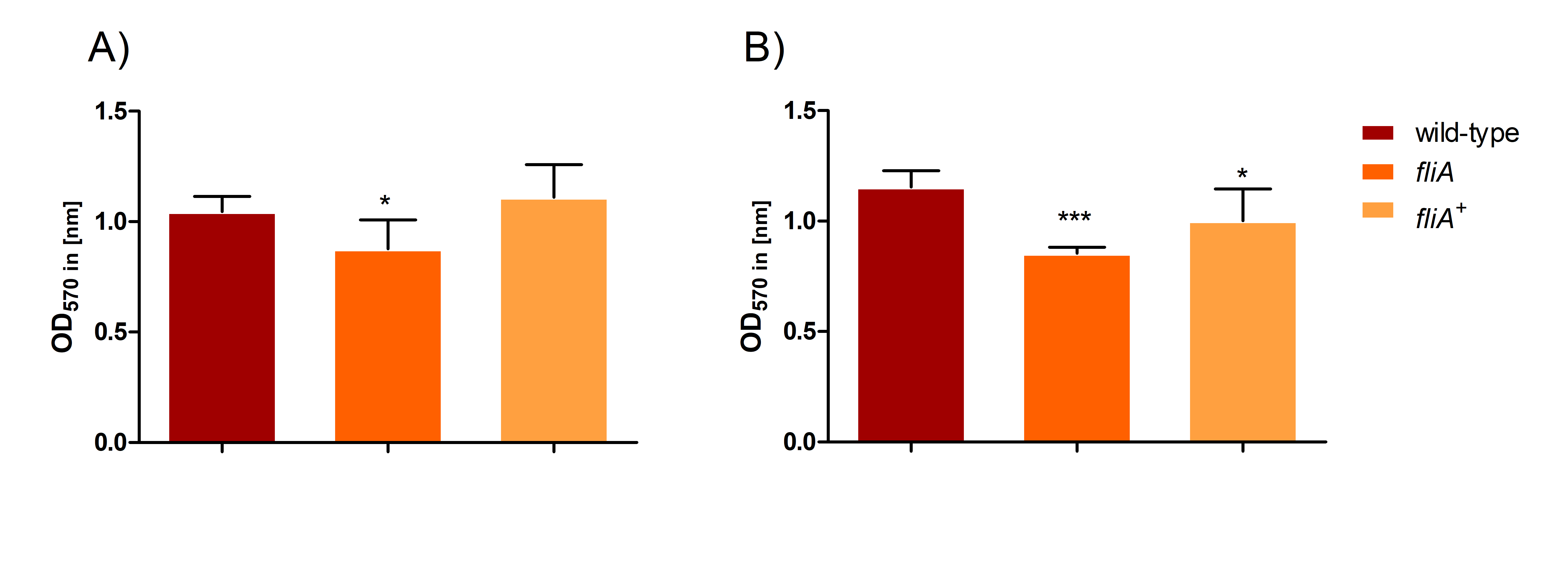

Supplement: Supplementary file 1 [file ijms-23-04560-s001.zip › Paszti et al_Supplemental Figure S2.tif]

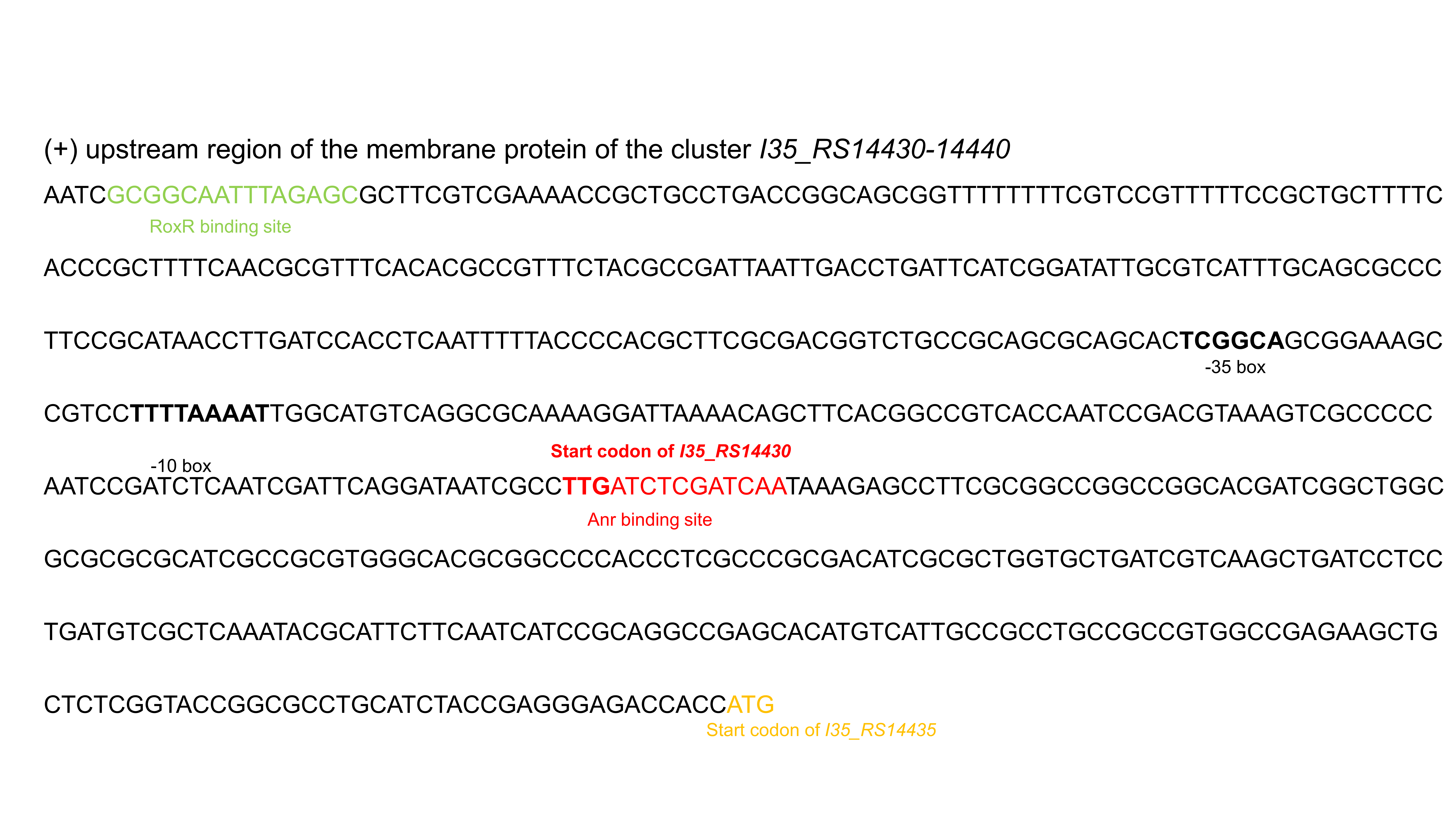

Supplement: Supplementary file 1 [file ijms-23-04560-s001.zip › Paszti et al_Supplemental Figure S3.png]
